# The Effect of Harvesting on the Composition of Essential Oils from Five Varieties of *Ocimum basilicum* L. Cultivated in the Island of Kefalonia, Greece

**DOI:** 10.3390/plants6030041

**Published:** 2017-09-18

**Authors:** Gerasimia Tsasi, Theofilos Mailis, Artemis Daskalaki, Eleni Sakadani, Panagis Razis, Yiannis Samaras, Helen Skaltsa

**Affiliations:** 1 Department of Food Technology, Technological Educational Institute (T.E.I.) of Ionian Islands, 28100 Argostoli, Greece; gtsasi@pharm.uoa.gr (G.T.); elenisakadani@hotmail.com (E.S.); p_razis@yahoo.gr (P.R.); ysamaras@teiion.gr (Y.S.); 2 Department of Pharmacognosy and Chemistry of Natural Products, School of Pharmacy, University of Athens, 15771 Athens, Greece; thema-89@hotmail.com (T.M.); artemisdask@hotmail.com (A.D.)

**Keywords:** basil varieties, essential oil, GC-MS, harvest, cluster analysis

## Abstract

Five varieties of *Ocimum basilicum* L. namely lettuce, cinnamon, minimum, latifolia, and violetto were separately cultivated in field and greenhouse in the island Kefalonia (Greece). The effect of successive harvesting to the essential oil content was evaluated. In total 23 samples of essential oils (EOs) were analyzed by GC-FID and GC-MS. Ninety-six constituents, which accounted for almost 99% of the oils, were identified. Cluster analysis was performed for all of the varieties in greenhouse and field conditions, in order to investigate the possible differentiation on the chemical composition of the essential oils, obtained between harvests during growing period. Each basil variety showed a unique chemical profile, but also the essential oil composition within each variety seems to be differentiated, affected by the harvests and the cultivation site.

## 1. Introduction

Basil (*Ocimum basilicum* L.) is an annual herb growing in several regions around the world. The genus *Ocimum* consists of more than 150 species whereof basil is cultivated in many countries as a major essential oil crop [1]. The species most commonly used as spices and medicinal herbs are *O. basilicum* L., *O. americanum* L. (syn. *O. canum* Sims.), *O. gratissimum* L., *O. kilimandscharicum* Guerke, *O. tenuiflorum* L. (syn. *O. sanctum* L.), and *O. africanum* Lour. (syn. *O.* × *citriodorum* Vis.), that is a hybrid of *O. basilicum* and *O. americanum* [2]. The taxonomy of the genus *Ocimum* is very complicated due to the occurrence of interspecific hybridization, polyploidy, aneuploidy, the existence of numerous botanical varieties, and chemotypes, as well as the many synonymous names [3,4,5]. Plants of the species *O. basilicum* have square stems, fragrant opposite leaves, and whorled flowers on spiked inflorescences [6]. *O. basilicum* L. is widely used in the culinary arts and in the food processing industry [7]. Traditionally, it has been used as medicinal plant for the treatment of headaches, coughs, diarrhea, constipation, warts, worms, and kidney malfunction [8]. A plethora of biological activities have been attributed to basil essential oils like antimicrobial, insecticidal, and recently was found to exhibit in vivo anti-malarial activity [9]. In addition, extracts from leaves and flowers can be used as aroma additives in food, pharmaceutical, and cosmetic industry [10]. Economical data clearly indicate the commercial importance of basil essential oil, since the world production is estimated to 1,200,000 € [8]. Basil essential oils contain a broad array of chemical compounds depending on variations in chemotypes, flower, and leaf colours, aroma and especially the origin of the plant [7]. The essential oil constituents vary among sweet basil cultivars, with linalool, methyl chavicol, eugenol, 1,8-cineole, geranial, neral, methyl cinnamate recognized as main components [11,12,13]. Several scientists have classified basil to different chemotypes according to the main components of the essential oil. Thereby, Marotti et al. (1996) divided essential oil from Italian basil varieties to three chemotypes “linalool”, “linalool-methyl chavicol”, and “linalool-eugenol” [14]. According to the plant origin, basils are grouped to European chemotype that has linalool and methyl chavicol as main components and Tropical chemotype having methyl chavicol as a main compound [15]. Concerning the cultivation of basil, the plants can be harvested one to three times during cropping season depending on the climate [16]. Generally, basil is harvested for the leaves that are sold fresh or dried. In the cases that basil is cultivated for dried leaves and extraction of essential oil, the plants are cut just prior the appearance of the flowers [17]. Scientific data concerning how harvest and number of harvests during the cultivation of aromatic plants affect essential oil quality and composition are lacking either in field or greenhouse conditions. To the best of our knowledge, so far Carlo et al. (2013) studied the effect of cut number in quality traits of sweet basil [18]. Also Zheljazkof et al. (2008) studied the effect of harvests in *O. basilicum* L. (cvs. German and Mesten) and *O. sanctum* L. (syn. *O. tenuiflorum* L.) (cv. Local) cultivated in Mississippi [9]. Taking into account that geographic position and the environmental characteristics of the habitat should be taken as factors affecting the chemical composition of the essential oils [19], the current study is the first including total chemical analysis of the essential oils from five basil varieties cultivated and harvested in the field and greenhouse in the island of Kefalonia, (Greece). The aim of our study is the chemical analysis of essential oils from the five varieties of basil considering also the cultivation site as well as how the ability of the plant to rejuvenate after successive cuttings during growing cycle, affects the obtained essential oil yield and composition. In order to simulate the natural growing conditions, successive cuttings were performed in every variety just before flowering stage, as done practically from aromatic plants’ growers. Moreover the cluster analysis of all basil varieties for greenhouse and field samples is given. 

## 2. Results and Discussion

### 2.1. Chemical Analysis

In the greenhouse conditions the essential oil (EO) content of varieties violetto, latifolia, and minimum increased in the second and/or third harvest, whereas in var. lettuce the second harvest produced less essential oil content when comparing to the first and third harvest where the essential oil content was in the same levels. Noteworthy is the fact, that in the field conditions, the essential oil content in the varieties of latifolia, minimum and lettuce decreased gradually after the successive harvests while in var. cinnamon sequential harvests didn’t affect overall essential oil yield (Table 1).

Results indicate that different cultivation conditions affect plant’s response to successive harvests and consequently the essential oil content, even of the same variety. As such controlled greenhouse conditions favor or have no impact to the total production of essential oil in the varieties latifolia, minimum, and lettuce when comparing to the field conditions. 

All obtained essential oils were analyzed with GC/FID and GC/MS. In total, 96 individual constituents were identified representing 98.0–99.9% of the total essential oil. The detailed chemical analysis of the essential oils of the greenhouse samples showed that the major constituents, in var. violetto, were linalool (22.5–26.5%) and *trans*-bergamontene (15.2–20.0%), followed by eugenol (4.5–11.9%). Variety latifolia has as major compounds linalool (17.1–35.6%), eugenol (10.1–23.3%) and *trans*-bergamontene (8.8–16.1%). In var. minimum linalool (27.4–28.3%) and eugenol (14.5–23.4%) were the main constituents while in var. lettuce linalool (21.0–23.6%), methyl chavicol (12.1–17.5%), *trans*-bergamontene (11.4–12.9%) and *epi*-α-cadinol (6.0–9.2%) (Table 2).

In the field conditions, the main constituents of var. latifolia were linalool (32.2–49.5%), eugenol (19.8–34.9%), *trans*-bergamontene (5.9–9.5%), and 1,8-cineole (4.2–6.7%). In var. minimun linalool (30.2–52.0%) and eugenol (28.8–36.0%), were the dominating compounds. Var. lettuce has as major components linalool (20.4–26.0%) and methyl-chavicol (45.4–54.1%). The latter constituent was dominating also in the essential oil of var. cinnamon in a range 61.2–75.1% among harvests (Table 3). Between harvests and cultivation sites, the main compounds of the essential oils displayed varying concentrations. The highest concentration for linalool (52.0%) was measured in var. minimum in the field cultivation, in the second harvest in contrast to what has been stated from Zheljazkof et al. (2008) that measures a higher content of linalool in the third cutting of *O. basilicum* varieties [9]. Interesting is the fact that eugenol showed the highest concentration in var. minimum in the first harvest in the field conditions. Noteworthy, from var. minimum methyl-chavicol is lacking or is present in small amounts. Given that methyl-chavicol has a structural resemblance to potential carcinogenic phenylpropanoids such as safrole, chemotypes rich in linalool are preferred for cultivation when used in food and perfume industries [15,20]. Methyl-chavicol showed the highest concentration (75.1%) in var. cinnamon in the field conditions from the first harvest followed by var. lettuce (54.1%) from the first harvest in the same conditions. In addition, it has been observed that the intensity of purple leaf colour is positively correlated in varieties rich in methyl chavicol [21]. However, in compliance with Liber et al. (2011), our results show that the biosynthesis of phenylopropanoids e.g., methyl chavicol, is not characteristic exclusively for the purple morfotypes. Also, green morfotypes (var. lettuce and cinnamon) are rich sources of methyl chavicol as well. [22].

Despite the observation that in the field conditions the total essential oil yield was decreasing after successive harvests the individual components, prevalent in basil, like linalool and eugenol seem to follow an opposite pattern. Linalool in var. latifolia was beyond 32.2% in all harvests and in the last harvest reached 49.5% while in var. minimum the percentage of linalool was in all harvests above 30.0% and in the second harvest reached 52.0% in the total essential oil concentration. Furtermore, although the concentrations of linalool in var. lettuce for both cultivation conditions were similar, compound methyl-chavicol showed a remarkable difference reaching 54.1% of the EO in the second harvest in the field conditions, whereas in the greenhouse conditions the higher concentration measured, was 17.5% in the first harvest (Table 2 and Table 3). These data suggest that changes in the environmental conditions can alter the biosynthesis of individual essential oils’ components. Ložienė et al. (2004) have reached similar conclusion while studying the influence of environmental and genetic factors in *Thymus pulegioides*’ essential oil [23]. Similar conclusions were reached also by Awadh Ali et al. (2017) while studying the EO content of *O. forskolei* and *Teucrium yemense* collected from different regions of Yemen [24]. In parallel, var. cinnamon grown in the field conditions has methyl-chavicol as major constituent reaching 75.1% of the EO content, in the first harvest (Table 3). It seems that the unstable field conditions, in combination with successive harvests affect and promote the metabolic pathway of L-phenylalanine and cinnamic acid [25], in var. latifolia, minimum and lettuce that leads to the production of methyl chavicol and eugenol. Moreover, the combination of the harvest stress with the unstable field conditions seems to promote the biosynthesis of individual components. In addition to this, the observed decrease in the EO yield in the field conditions during harvests can be attributed to the partial evaporation of the essential oil from the plant surface.

### 2.2. Statistical Analysis 

The statistical analysis of our results confirmed the laborious interaction between the essential oil composition, different varieties, and cultivation conditions. After thorough study of the cluster analyses, it is obvious that during the growing period, varieties can alter the composition of individual components of the essential oil by shifting their chemotype [23]. 

**Figure 1:**
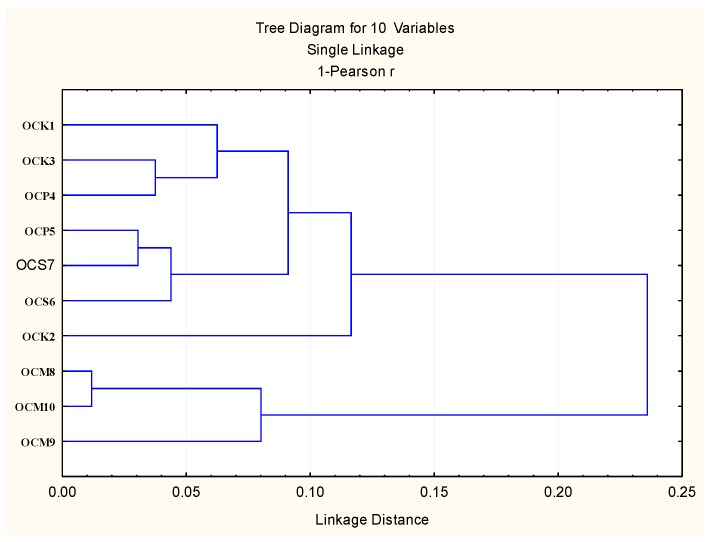
Bidimensional dendrogram representing the similarity in the main components of the essential oils, between 10 harvests of 4 basil varieties growing in the greenhouse conditions.

**Figure 2:**
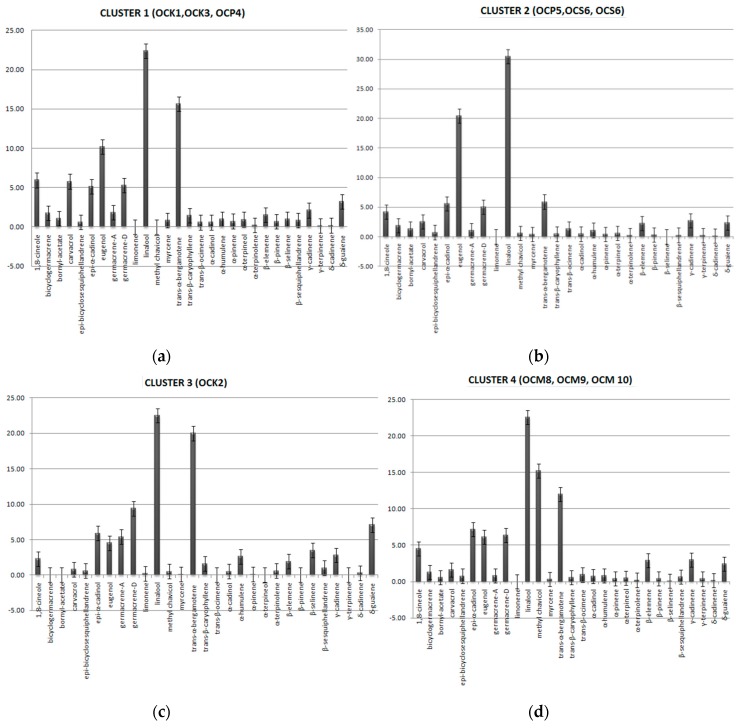
Clusters formatted according to the dominant compounds identified, in varieties violetto, latifolia, minimum and lettuce, cultivated in the greenhouse conditions. (**a**) Dominant compounds in var. violetto (1st and 3rd harvest) and var. latifolia (1st harvest); (**b**) Dominant compounds in var. latifolia (2nd harvest) and var. minimum (1st and 2nd harvest); (**c**) Dominant compounds in var. violetto (2nd harvest); (**d**) Dominant compounds in var. lettuce (1st, 2nd and 3rd harvest).

Taking into account the EO content similarities between harvests for the varieties cultivated in the greenhouse, four clusters were formed (Figure 1). Cluster 1 consists of samples OCK1, OCK3, and OCP4, with linalool, *trans*-α-bergamontene, and eugenol as the dominant components. Cluster 2 consists of samples OCP5, OCS6, and OCS7, with linalool and eugenol as the dominant components. Cluster 3 is “simplicifolious” and consists of OCK2 with major constituents linalool and *trans*-α-bergamontene. Finally, cluster 4 consists of OCM8, OCM9, and OCM10 with major components linalool, methyl chavichol, and *trans*-α-bergamontene (Figure 2).

**Figure 3:**
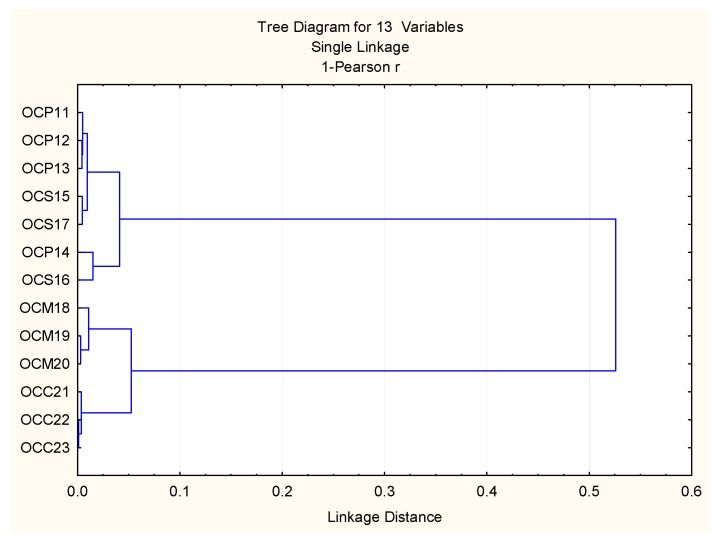
Bidimensional dendrogram representing the similarity in the main components of the essential oils, between 13 harvests of 4 basil varieties growing in the field conditions.

**Figure 4:**
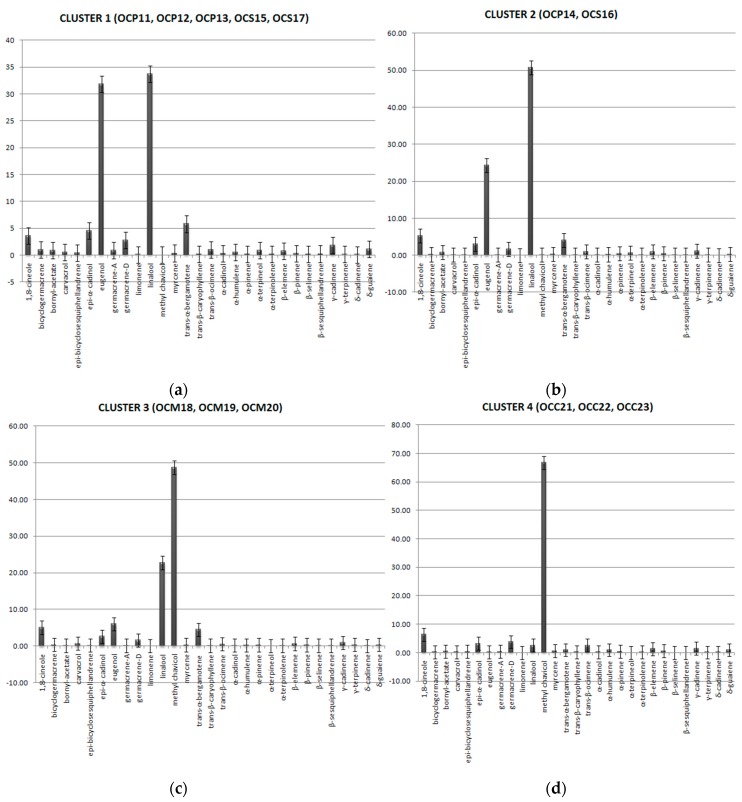
Clusters formatted according to the dominant compounds identified in varieties latifolia, minimum, lettuce and cinnamon, cultivated in the field conditions. (**a**) Dominant compounds in var. latifolia (1st, 2nd and 3rd harvest) and var. minimum (1st and 3rd harvest); (**b**) Dominant compounds in var. latifolia (4th harvest) and var. minimum (2nd harvest); (**c**) Dominant compounds in var. lettuce (1st , 2nd, and 3rd harvest); (**d**) Dominant compounds in var. cinnamon (1st, 2nd and 3rd harvest).

Cultivation conditions in the field defined the dominant constituents for the formation of four clusters in the dendrogram (Figure 3). The similarities observed between harvests and varieties characterized the clusters as: Cluster 1 consisting of samples OCP11, OCP12, OCP13, OCS15, and OCS17 with linalool, eugenol, and *trans*-α-bergamontene as major components. Cluster 2 consists of OCP14 and OCS16 with linalool and eugenol as major components. Cluster 3 consists of OCM18, OCM19, and OCM20, with methyl chavicol and linalool as major components. Finally, cluster 4 is “simplicifolious” and consists of samples OCC21, OCC22, and OCC23, with methyl-chavicol as the dominant component (Figure 4).

Considering that in both field and greenhouse the conditions were same for all of the varieties, the observed variations can be attributed to the ability of each variety to react to the stressing factor of harvesting. The grouping obtained for the examined basil varieties in greenhouse and field conditions, provides significant data about the effect of harvesting and cultivation site in the chemical composition of basil essential oils. A comparison of Cluster 1 between field and greenhouse conditions reveals that in var. latifolia, between harvests, shifts are observed in the chemotype by alterations in the biosynthesis of essential oils’ components. These results are in compliance with the study of Božović et al. (2017), in EOs obtained from *Calamintha nepeta* (L.) Savi subsp. *glandulosa,* indicating that the main compounds were stable, but the ratio between them varied greatly according to the growth stage [26]. Especially in var. lettuce it is obvious that apart from the harvest factor, the cultivation site also affects the chemical profile of the obtained EO. Observation of cluster 3 from field cultivation and cluster 4 from greenhouse cultivation reveals a different chemical profile in the EOs obtained for the same variety. As such, these results imply that the cultivation site should also be considered as factor affecting the composition of EOs, apart from the number of harvests. The variations of the chemical profile of the five basil varieties are in accordance with the results of Verma et al. (2013) where different *Ocimum* populations belonging to the “basilicum” group are distributed to different clusters [27]. The alteration in the chemical profile of the essential oils obtained from *Foeniculum vulgare* Miller between harvests was also indicated by Garzoli et al. (2017). Researchers showed that while *o*-cymene and *a*-phellandrene were dominating compounds in the first two harvests, EO from the last harvest was characterized by a high content of methyl-chavichol (estragole) [28]. Cluster analysis has been proved as a useful tool in the attempt of an evaluation of chemotypes among essential oil bearing plants. However, in *O. basilicum’s* case, the numerous synonymous names, varieties, and inerspecific hybridization along with the several factors that affecting essential oils’ composition make this process extremely difficult [29]. 

The obtained data can serve as a guide for cultivation conditions and the harvesting of basil varieties in this geographical region in order to obtain maximum essential oil yield along with certain metabolites or mixtures of them in the composition of the essential oil. Comparing to the literature data about basil chemical composition, current results are in accordance about the yield of essential oil produced. Additionally, they are very promising concerning the composition of them to important constituents like linalool, eugenol, and methyl chavicol. Environmental conditions, like temperature, humidity, and soil conditions, in the island of Kefalonia affect the chemical profile of the cultivated basil varieties and favor the production of bioactive compounds like linalool [30]. Practically, this implies that essential oils from aromatic plants cultivated in the climatic conditions of Kefalonia have the quality characteristics needed for commercial exploitation.

## 3. Materials and Methods

### 3.1. Cultivation Conditions and Plant Material

**Figure 5:**
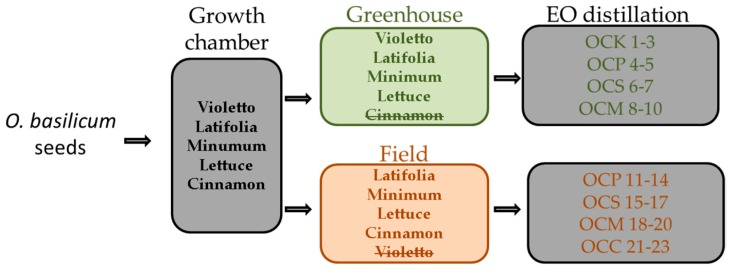
Diagrammatic presentation of the cultivation of basil seeds belonging to five varieties in field and greenhouse, along with the relevant obtained essential oils (EOs).

Certified *Ocimum basilicum* L. (basil) seeds, belonging to varieties lettuce, cinnamon, minimum, latifolia, and violetto, were purchased from the local market. Basil seeds of the five varieties were sown into sowing boxes filled with universal soil for sowing seeds in an environmentally controlled growth chamber. Environmental conditions were: photoperiod 12/12, air temperature 18/24 °C, and air relative humidity 50/70% (day/night). When the plants formed true leaves were transplanted into small plastic pots. When they reached 12–15 cm height, they were transplanted to their final position either into plastic 20 L pots in the greenhouse or directly to the land in the experimental field. For each variety 20 plants were planted in each cultivation site. For the field conditions, basil transplants were planted into rows with 0.50 m in-row and 1.00 m between row spacing, covering a total area of 15 m^2^. Plants were irrigated every second day with an irrigation tape providing 5–7 L of water, in both field and greenhouse conditions. During the growing period, no pests or diseases were observed apart from the inability of var. cinnamon to grow in the greenhouse conditions and that var. violetto failed to grow in the field conditions, thus only the results from one cultivation site are given. Harvesting of the plants was performed almost two months after transplantation, prior to flowering stage [17]. The field cultivation conducted in the experimental field of the Department of Food Technology (Technological Educational Institute of Ionian Islands) in the island of Kefalonia (Figure 5). Plant material was hand harvested by removing the apical portion of the herbage 20 cm above ground and leaving 1–2 internodes in order to promote rejuvenation from the sleeping buds. For each variety and cultivation site, 2–3 harvests were performed depending on the variety and its ability to regenerate stems and flowers. Practically, this includes a period of 30–40 days between each cutting. In every collection, plant material was collected the same time of the day in order to eliminate day variations to the yield of essential oil, transferred to the laboratory and fresh weight was measured, and the essential oil yield to fresh weight was calculated. In total, 23 samples were collected extracted and analysed, namely 10 essential oils obtained from 4 four different varieties cultivated in the greenhouse, and 13 essential oils obtained from four varieties cultivated in the field (Table 1). The plant materials have been deposited in the herbarium of the Department of Food Technology (Technological Educational Institute of Ionian Islands) under the number 737. 

### 3.2. Essential Oil Distillation

The collected plant material from the five varieties was separately subjected to hydro distillation in a modified Clevenger-type apparatus according to the Hellenic Pharmacopoeia [31]. Each plant material sample (about 2.0 kg) was extracted for 3 h. The essential oil content (% *v/w*) was estimated on a fresh weight basis. The oil samples were dehydrated over anhydrous Na_2_SO_4_. The obtained essential oils were of yellowish colour and a pleasant odor, and were deposited in vials at −20 °C prior to further chemical analyses.

### 3.3. GC Analysis

Quantification was performed using gas-chromatography coupled with flame ionization detection (GC-FID). Analyses were carried out on a Perkin Elmer Clarus 500 gas chromatograph with FID, fitted with a fused silica Rtx-5 MS capillary column (30 m × 0.25 mm (i.d.), film thickness: 0.25 μm). The column temperature was programmed from 60 °C to 250 °C at a rate of 3 °C/min. The injector and detector temperatures were programmed at 230 °C and 280 °C, respectively. 0.5 µL of each sample were diluted in 500 µL GC grade n-pentane and 2 μL of the obtained solution was further injected in the GC apparatus. Identification of constituents was achieved by calculating the arithmetic indices relative to linear alkanes from C_9_–C_23_ and comparing with data from GC-MS identifications.

### 3.4. GC-MS Analysis

GC-MS analyses were performed on a Hewlett-Packard 5973-6890 system operating in EI mode (70 eV) equipped with a split/splitless injector (220 °C), a split ratio 1/10, using a fused silica HP-5 MS capillary column (30 m × 0.25 mm (i.d.), film thickness: 0.25 μm). The temperature program for HP-5 MS column was from 60 °C (5 min) to 280 °C at a rate of 4 °C/min. Helium was used as a carrier gas at a flow rate of 1.0 mL/min. Injection volume for all samples, diluted as previously described, was 2 μL.

### 3.5. Identification of Components

Retention indices for all compounds were determined according to the Van der Dool approach [32] with reference to a homologous series of normal *n*-alkanes from C_9_ to C_23_. The identification of the components was based on a comparison of their mass spectra with those of Wiley and NBS Libraries and those described by Adams (2007), as well as by comparison of their retention indices with literature data [33]. Component relative percentages were calculated based on GC-FID peak areas without using correction factors.

### 3.6. Statistical Analysis

Data obtained from the chemical analysis of the essential oils from all varieties were analyzed by Multivariate analysis method using Statistica software version 7.0 Statsoft Company. Cluster analyses were performed based on the similarity between harvests from all varieties and their constituent distribution. These analyses were performed on complete data sets. The unweighted pair-group average linkage clustering method based on Pearson distances was used to measure the similarities between each measured unit.

## Figures and Tables

**Table 1:** Samples from *O. basilicum* varieties and essential oil yield.

*O. basilicum* L. Varieties	Harvest	Cultivation Site	EO (% Fresh Weight)
*O. basilicum* L. var. *violetto*	**OCK1**	greenhouse	0.017
	**OCK2**	greenhouse	0.04
	**OCK3**	greenhouse	0.08
*O. basilicum* L. var. *latifolia*	**OCP4**	greenhouse	0.026
	**OCP5**	greenhouse	0.14
*O. basilicum* L. var. *minimum*	**OCS6**	greenhouse	0.033
	**OCS7**	greenhouse	0.08
*O. basilicum* L. var. *lettuce*	**OCM8**	greenhouse	0.10
	**OCM9**	greenhouse	0.04
	**OCM10**	greenhouse	0.15
*O. basilicum* L. var. *latifolia*	**OCP11**	field	0.18
	**OCP12**	field	0.20
	**OCP13**	field	0.12
	**OCP14**	field	0.08
*O. basilicum* L. var. *minimum*	**OCS15**	field	0.11
	**OCS16**	field	0.04
	**OCS17**	field	0.036
*O. basilicum* L. var *lettuce*	**OCM18**	field	0.12
	**OCM19**	field	0.07
	**OCM20**	field	0.08
*O. basilicum* L. var. *cinnamon*	**OCC21**	field	0.21
	**OCC22**	field	0.21
	**OCC23**	field	0.20

**Table 2:** Chemical composition of the essential oils from *O. basilicum* L. varieties, violetto, latifolia, minimum and lettuce, cultivated in the greenhouse conditions.

	Compound	RI ^a^	OCK1	OCK2	OCK3	OCP4	OCP5	OCS6	OCS7	OCM8	OCM9	OCM10
1	α-thujene	923	-	-	tr	-	-	tr	tr	tr	tr	tr
2	α-pinene	932	0.9	0.1	0.4	0.8	0.2	0.8	0.3	0.5	0.3	0.4
3	camphene	946	-	-	tr	0.1	0.1	0.1	0.1	0.1	tr	tr
4	sabinene	968	0.3	0.1	0.3	0.4	0.2	0.1	0.1	0.2	0.1	0.2
5	β-pinene	973	0.7	-	0.6	0.7	0.4	0.3	0.2	0.5	0.2	0.5
6	myrcene	984	1.0	0.1	0.7	0.8	0.5	0.4	0.3	0.3	0.2	0.4
7	α-phellandrene	1001	-	-	0.2	-	-	-	0.1	-	0.1	0.1
8	δ-3-carene	1004	0.3	-	-	0.4	0.1	0.4	tr	0.2	0.1	-
9	α-terpinene	1011	-	-	0.1	0.1	-	0.1	0.1	0.1	0.1	0.1
10	limonene	1019	-	0.2	-	-	-	-	-	-	-	-
11	p-cymene	1017	-	-	-	-	-	-	0.1	-	-	-
12	1,8-cineole	1027	6.6	2.3	6.3	4.9	7.1	2.7	2.7	5.2	3.5	4.8
13	*trans*-β-ocimene	1043	-	-	tr	1.7	1.7	1.1	1.3	0.9	0.8	1.1
14	γ-terpinene	1051	0.1	-	0.2	0.1	0.1	0.2	0.4	0.3	0.4	0.4
15	α-terpinolene	1078	0.1	0.6	0.1	0.4	0.2	0.3	0.2	0.2	0.2	0.2
16	linalool	1100	26.5	22.5	23.5	17.1	35.6	27.4	28.3	21.0	23.1	23.6
17	allo-ocimene	1133	-	-	-	-	-	-	-	tr	-	-
18	*trans*-epoxy-ocimene	1134	-	-	-	0.2	0.4	0.1	-	-	-	-
19	camphor	1142	-	-	tr	0.2	0.3	0.3	0.3	0.4	0.6	0.5
20	borneol	1166	-	-	0.2	0.5	0.8	0.3	0.3	-	-	-
21	terpinen-4-ol	1172	0.2	0.2	0.2	0.2	0.2	0.5	1.2	-	2.0	1.9
22	α-terpineol	1184	0.7	-	1.4	0.7	1.3	0.4	-	1.5	-	-
23	methyl chavicol	1193	-	0.5	-	-	-	-	1.9	17.5	12.1	16.0
24	myrtenal	1205	-	-	-	-	-	-	-	-	tr	-
25	octyl acetate	1207	0.2	-	0.2	0.4	0.1	0.2	0.2	-	-	-
26	endo-fenchyl acetate	1213	0.7	-	0.4		-	-	-	-	-	-
27	neral	1231	-	-	-	-	-	0.1	-	-	-	-
28	chavicol	1245	-	-	-	-	-	-	-	1.0	0.3	0.8
29	geraniol	1247	-	-	-	-	-	-	0.9	-	-	-
30	*trans*-anethole	1280	-	-	0.7	-	-	-	0.2	-	0.3	-
31	bornyl-acetate	1282	-	-	-	3.2	1.4	1.5	1.1	0.7	0.5	0.6
32	carvacrol	1296	10.0	0.8	2.9	4.4	0.7	4.3	2.7	2.3	1.1	1.5
33	α-cubebene	1334	-	-	0.1	0.1	-	0.1	tr	0.1	-	tr
34	eugenol	1356	8.6	4.5	11.9	10.1	23.3	14.5	23.4	7.2	2.1	9.1
35	α-copaene	1369	0.2	0.2	-	tr	0.1	-	-	0.3	0.2	-
36	β-patchoulene	1372	-	-	-	-	-	-	-	0.1	0.1	0.1
37	β-cubebene	1375	-	-	-	-	-	tr	0.1	-	-	-
38	β-elemene	1387	1.4	1.9	1.9	1.3	0.9	3.6	2.3	2.6	3.8	2.4
39	methyl eugenol	1399	0.1	-	0.4	0.2	0.1	0.1	0.1	0.1	0.1	0.1
40	*cis*-α-bergamotene	1398	-	-	-	-	0.1	-	0.1	-	-	-
41	*trans*-β-caryophyllene	1409	1.3	1.6	1.9	1.1	0.2	0.8	0.4	0.6	0.6	0.4
42	β-gurjunene	1417	-	0.7	-	-	-	0.5	0.3	-	-	-
43	*trans*-α-bergamotene	1432	15.6	20.0	15.2	16.1	8.8	3.8	5.1	11.4	12.9	11.7
44	aromadendrene	1437	0.2	0.3	0.3	0.4	0.2	0.6	0.1	0.4	0.5	0.4
45	*cis*-muurola-3,5-diene	1439	-	-	-	-	0.3	0.6	0.5	-	-	-
46	*trans*-β-farnesene	1446	0.3	0.4	-	1.3	-	-	-	-	-	0.4
47	α-humulene	1448	1.1	2.6	1.4	0.4	0.9	1.5	0.9	0.4	1.4	0.6
48	epi-bicyclosesquiphellandrene	1453	0.4	0.6	0.5	0.9	0.4	1.0	0.8	0.7	0.9	0.7
49	allo-aromadendrene	1459	-	-	0.2	0.2	-	0.2	0.1	tr	-	-
50	α-amorphene	1460	-	-	0.3	-	-	-	-	tr	-	-
51	germacrene-D	1476	3.7	9.4	5.4	6.7	3.6	7.0	4.5	5.4	8.2	5.4
52	β-selinene	1475	2.9	3.5	-	-	-	-	-	0.2	-	-
53	bicyclogermacrene	1486	-	-	3.0	2.2	1.1	2.5	2.0	1.3	1.2	1.3
54	δ-guaiene	1496	2.7	7.1	3.8	3.1	1.2	3.8	2.1	2.3	3.3	1.7
55	germacrene-A	1499	2.6	5.4	1.5	1.4	1.1	1.1	1.0	0.8	1.0	0.7
56	γ-cadinene	1506	1.9	2.8	1.7	2.7	1.6	3.2	3.4	2.7	3.5	2.8
57	β-sesquiphellandrene	1514	0.7	1.0	0.9	0.9	0.4	0.2	0.2	0.6	0.7	0.6
58	δ-cadinene	1516	0.2	0.3	0.1	0.2	0.1	0.2	0.1	0.1	0.2	0.1
59	α-cadinene	1520	-	-	0.1	0.1	0.2	0.1	0.1	0.1	tr	0.1
60	*trans*-γ-bisabolene	1530	0.2	-	-	0.1	-	-	-	0.1	0.1	0.1
61	γ-cuprenene	1535	0.1	0.2	0.1	0.1	-	-	-	tr	0.1	tr
62	*trans*-cadina-1,4-diene	1534	-	-	0.1	0.1	-	0.1	-	0.1	0.2	tr
63	germacrene B	1548	-	-	-	0.1	-	-	-	-	-	-
64	*trans*-nerodilol	1543	-	-	0.4	-	-	0.2	0.1	0.1	0.1	0.2
65	maaliol	1550	-	1.5	0.5	-	-	-	-	0.1	0.9	0.2
66	caryophyllene oxide	1557	-	-	-	-	-	-	-	tr	-	-
67	spathulenol	1562	-	-	0.1	0.1	-	-	tr	tr	0.1	tr
68	viridiflorol	1595	0.5	0.7	0.9	1.1	-	-	-	0.9	1.1	0.8
69	1,10-di-epi-cubenol	1596	-	-	-	-	0.4	1.0	0.8	-	-	-
70	epi-α-cadinol	1638	3.7	5.9	5.0	6.7	3.2	7.2	6.3	6.2	9.2	6.0
71	vulgarone B	1641	-	-	0.2	-	-	-	-	-	-	-
72	α-eudesmol	1651	-	-	0.1	-	-	-	-	-	-	-
73	α-cadinol	1652	0.2	0.5	0.7	0.8	0.2	0.7	0.5	0.7	0.9	0.6
74	7-epi-α-eudesmol	1659	-	-	-	-	-	-	-	tr	-	-
75	α-bisabolol	1685	0.6	-	0.2	0.2	-	0.1	0.1	0.1	0.1	0.1
76	germacrone	1691	-	-	-	0.3	-	-	-	-	-	-
77	*cis*-farnesol	1692	-	-	0.3	-	-	-	-	0.1	0.2	0.1
78	β-sinensal	1693	-	-	0.4	-	-	0.2	-	0.2	0.2	0.1
79	mint sulfide	1733	-	-	tr	0.1	-	0.1	-	tr	-	-
80	neo phytadiene	1838	-	-	-	0.8	-	-	-	-	-	-
81	nonadecane	1900	-	-	-	tr	-	-	-	-	-	-
82	eicosane	2000	-	-	-	0.4	-	-	-	-	-	-
83	heneicosane	2100	-	-	-	0.2	-	-	-	-	-	-
84	docosane	2200	-	-	-	0.2	-	-	-	-	-	-
85	tricosane	2300	-	-	-	0.1	-	-	-	-	-	-
86	tetracosane	2400	-	-	-	-	-	tr	-	-	-	-
87	pentacosane	2500	-	-	-	-	-	0.2	-	tr	-	-
88	hexacosane	2600	-	-	-	-	-	tr	-	-	-	-
89	heptacosane	2700	0.3	0.5	0.1	-	-	0.2	-	0.1	-	-
90	nonacosane	2900	0.3	0.5	0.1	-	-	-	-	0.1	-	-
91	triacontane	3000	0.2	0.3	0.1	-	-	-	-	0.1	-	-
**Total**			**98.3**	**99.8**	**98.3**	**98.0**	**99.8**	**97.1**	**98.5**	**99.2**	**99.9**	**99.9**

^a^ Retention indices were calculated against C9-C23 *n*-alkanes on the HP 5MS capillary column.

**Table 3:** Chemical composition of the essential oils from *O. basilicum* L. varieties latifolia, minimum, lettuce and cinnamon, cultivated in the field conditions.

	Compound	RI *^a^*	OCP11	OCP12	OCP13	OCP14	OCS15	OCS16	OCS17	OCM18	OCM19	OCM20	OCC21	OCC22	OCC23
1	α-thujene	922	-	-	-	-	-	-	tr	tr	tr	tr	tr	tr	tr
2	α-pinene	931	0.2	0.2	0.2	0.5	tr	0.3	0.3	0.3	0.3	0.2	0.2	0.5	0.5
3	Camphene	946	0.1	tr	tr	0.1	tr	0.1	0.1	tr	tr	tr	tr	0.2	0.2
4	sabinene	967	0.2	0.2	0.2	0.2	tr	0.2	0.1	0.1	0.2	0.1	0.2	0.4	0.4
5	β-pinene	974	0.3	0.3	0.3	0.5	0.1	0.3	0.3	0.3	0.3	0.3	0.3	0.7	0.8
6	myrcene	984	0.3	0.3	0.5	0.7	0.1	-	0.5	0.2	0.3	0.3	0.4	0.7	0.8
7	3-octanol	988	-	-	-	-	-	-	-	-	-	tr	-	-	-
8	n-decane	1000	-	-	-	-	-	-	-	-	-	0,1	-	-	-
9	α-phellan-drene	1002	-	-	-	-	tr	-	tr	-	-	tr	tr	0.1	0.1
10	δ-3-carene	1004	tr	tr	0.1	0.1	tr	-	tr	tr	0.1	-	-	-	-
11	α-terpinene	1013	-	-	tr	0.1	0.1	-	tr	0.1	0.1	0.1	tr	0.1	0.1
12	p-cymene	1019	-	-	-	-	0.1	-	tr	-	-	0.1	-	-	-
13	limonene	1022	-	-	-	-	0.1	-	0.2	-	-	-	-	-	-
14	1,8-cineole	1026	4.2	4.2	5.1	6.7	1.6	3.8	2.7	5.7	4.8	4.6	4.8	6.8	7.6
15	*cis*-β-ocimene	1035	-	-	-	-	-	-	-	-	tr	-	-	-	-
16	*trans*-β-ocimene	1043	1.4	1.1	0.9	0.6	0.8	1.4	0.9	0.7	0.5	0.4	1.9	3.0	2.9
17	γ-terpinene	1053	0.1	0.1	0.1	tr	0.2	0.2	0.2	0.3	0.3	0.4	0.1	0.1	0.1
18	*cis*-sabinene hydrate	1064	-	-	-	-	-	-	-	-	-	tr	-	tr	tr
19	α-terpinolene	1081	0.2	0.2	0.1	0.2	0.2	0.1	0.3	0.1	0.1	0.1	0.1	0.3	0.3
20	fenchone	1081	-	-	-	-	-	-	-	-	-	tr	-	-	-
21	linalool	1100	32.2	34.0	35.5	49.5	36.8	52.0	30.2	26.0	21.8	20.4	5.7	0.6	1.3
22	allo-ocimene	1132	-	-	-	-	-	-	-	tr	tr	tr	-	-	-
23	*trans*-epoxy-ocimene	1134	0.3	0.3	0.1	tr	0.1	-	tr	-	-	-	tr	0.1	0.1
24	camphor	1141	0.3	0.3	0.1	0.1	0.3	0.2	0.3	0.3	0.2	0.3	0.6	2.1	1.9
25	borneol	1165	0.6	0.6	0.5	0.4	0.4	0.2	0.4	-	-	0.2	-	-	tr
26	terpinen-4-ol	1173	0.1	0.1	0.2	0.1	1.2	1.1	0.8	-	tr	2.1	-	-	-
27	α-terpineol	1184	0.7	1.0	1.0	0.8	0.8	0.5	0.9	-	-	-	-	-	-
28	methyl chavicol	1186	-	-	-	-	-	0.3	-	45.4	54.1	46.7	75.1	60.2	64.9
29	octyl acetate	1208	0.1	0.1	0.1	0.2	0.2	0.1	0.3	-	-	-	-	-	-
30	nerol	1224	-	-	-	-	-	-	0.1	tr	tr	tr	-	-	-
31	citronellol	1225	-	-	-	-	-	-	0.1	-	-	tr	-	-	-
32	neral	1234	-	-	-	-	-	-	0.1	-	-	-	-	-	-
33	carvone	1239	-	-	-	-	-	-	0.1	-	tr	-	tr	-	-
34	chavicol	1243	-	-	-	-	-	-	-	0.2	0.2	0.5	tr	0.1	0.1
35	geraniol	1245	-	-	-	-	0.6	0.7	1.5	-	-	-	-	-	-
36	citronellyl formate	1277	-	-	-	-	-	-	-	-	-	-	-	-	tr
37	*trans*-anethole	1280				-	-	-	-	tr	tr	-	-	tr	tr
38	bornyl acetate	1289	0.8	0.8	0.6	1.2	0.6	0.3	1.3	0.1	0.1	0.2	0.2	0.6	0.5
39	carvacrol	1298	1.0	0.4	0.3	tr	0.7	0.3	0.3	0.7	1.1	0.2	0.2	0.4	0.2
40	δ-elemene	1336	-	-	-	-	-	-	-	-	-	-	tr	tr	tr
41	carvyl acetate	1333	-	-	-	-	-	-	-	-	-	-	-	tr	tr
42	α-cubebene	1341	-	-	-	-	-	-	0.1	-	-	0.1	-	0.1	0.1
43	eugenol	1356	26.9	32.2	34.9	19.8	36.0	28.8	28.9	10.0	2.5	5.6	0.1	0.1	0.2
44	neryl acetate	1359	-	-	-	-	0.2	-	-	-	-	-	-	-	-
45	α-copaene	1369	0.1	-	-	-	-	tr	-	-	tr	0.1	tr	0.2	0.2
46	geranyl acetate	1378	-	-	-	-	-	-	0.8	-	-	-	-	-	-
47	β-bourbonene	1386	-	-	-	-	-	-	-	tr	tr	0.1	0.1	0.2	0.1
48	β-cubebene	1387	-	-	-	-	-	-	-	-	-	tr	-	-	-
49	β-elemene	1391	1.0	-	tr	0.7	1.9	1.1	1.1	0.5	0.6	0.8	0.9	1.9	1.3
50	methyl-eugenol	1402	0.1	0.2	0.2	0.1	0.1	-	0.2	tr	tr	0.1	0.1	0.5	0.5
51	α-gurjunene	1398	-	-	-	-	-	-	-	-	-	-	-	tr	tr
52	*cis*-α-bergamotene	1401	0.1	-	-	-	-		-	tr	tr	0.1	-	tr	tr
53	*trans*-β-caryophyllene	1415	0.2	0.2	0.2	0.2	0.2	0.1	0.2	0.1	0.1	0.1	0.1	0.4	0.3
54	β-copaene	1414	-	-	-	-	-	-	-	-	-	0.1	-	0.1	0.1
55	β-gurjunene	1420	0.4	-	-	-	-	-	0.1	-	-	-	tr	0.2	-
56	*trans*-α-bergamotene	1431	9.5	7.6	6.5	5.9	2.2	2.1	3.1	4.0	5.1	4.4	0.4	1.0	1.5
57	α-guaiene	1427	-	-	-	-	-	-	-	-	-	-	0.1	0.4	-
58	aromadendrene	1435	0.2	-	-	0.1	-	-	0.1	tr	0.1	0.1	tr	tr	-
59	*cis*-muurola-3.5-diene	1439	0.3	0.2	0.2	-	0.2	-	0.3	-	-	-	-	-	-
60	*trans*-β-farnesene	1445	-	-	0.2	0.2	-	-	0.2	-	0.1	-	-	-	-
61	α-humulene	1448	0.8	0.8	0.2	0.2	0.5	0.2	0.3	0.2	0.1	0.4	0.5	1.4	1.0
62	epi-bicyclosesquiphellandrene	1454	0.5	0.4	0.3	0.2	0.4	-	0.6	0.1	0.1	0.3	0.1	0.5	0.4
63	allo-aromadendrene	1456	-	-	2.6	tr	-	0.1	tr	-	-	-	-	-	-
64	α-amorphene	1460	-	-	-	-	-	-	-	-	-	0.1	-	0.1	0.1
65	germacrene-D	1475	4.2	3.5	0.1	1.9	3.0	1.5	2.8	1.2	1.6	2.0	3.2	5.0	3.2
66	β-selinene	1479	-	0.1	0.8	0.1	tr	-	0.1	0.2	tr	0.1	-	-	-
67	bicyclogermacrene	1486	1.0	0.9	0.9	0.4	0.9	-	1.2	0.2	0.3	0.5	0.3	0.3	0.2
68	δ-guaiene	1493	1.4	1.3	0.4	0.5	1.4	-	1.3	0.1	0.3	0.6	0.5	1.4	1.0
69	germacrene-A	1498	1.2	0.7	1.7	0.2	0.5	-	0.4	-	0.1	0.3	0.2	0.5	0.3
70	γ-cadinene	1505	1.9	1.6	0.3	1.5	2.0	0.9	3.2	0.7	0.9	1.2	0.9	1.9	1.6
71	*trans*-calamenene	1506	-	-	-	-	tr	-	0.2	-	tr	-	tr	0.1	-
72	β-sesquiphellandrene	1508	0.5	0.4	tr	0.2	0.1	-	0.1	0.1	0.1	0.2	-	-	-
73	δ-cadinene	1514	0.2	0.1	tr	tr	tr	-	0.1	-	tr	0.1	tr	0.1	0.1
74	α-cadinene	1520	-	tr	-	tr	tr	-	-	-	tr	tr	tr	0.2	0.1
75	γ-cuprenene	1528	-	0.1	-	-	-	-	tr	-	-	-	-	tr	tr
76	*trans*-γ-bisabolene	1530	0.1	-	-	-	-	-	-	-	tr	-	tr	tr	tr
77	germacrene B	1551	0.7	0.5	-	-	-	-	-	-	-	-	-	-	-
78	*trans*-nerolidol	1557	-	-	-	tr	0.1	-	0.1	tr	tr	0.1	tr	tr	tr
79	maaliol	1565	-	-	-	-	-	-	-	tr	tr	0.1	tr	0.2	0.1
80	spathulenol	1571	-	tr	-	0.1	-	-	tr	-	tr	tr	-	0.1	tr
81	caryophyllene-oxide	1585	-	-	-	-	-	-	-	-	-	tr	-	tr	tr
82	viridiflorol	1592	-	-	-	-	-	-	0.2	0.1	0.2	0.4	0.2	0.6	0.5
83	guaiol	1600	-	-	-	-	-	-	-	-	-	-	-	tr	-
84	1,10-di-*epi*-cubenol	1616	0.5	0.5	0.5	0.5	0.6	-	1.0	-	-	-	-	-	-
85	epi-α-cadinol	1637	4.5	3.8	3.7	4.4	4.1	1.8	6.6	1.8	2.9	3.2	2.3	4.1	3.1
86	α-cadinol	1653	0.3	0.3	0.3	0.2	0.2	-	0.5	0.1	0.1	0.3	0.1	0.4	0.3
87	allo himachalol	1654	-	-	-	-	-	-	-	-	-	-	-	tr	tr
88	β-bisabolol	1681	-	-	-	-	-	-	0.2	-	-	-	-	-	-
89	α-bisabolol	1683	-	0.1	tr	0.1	0.1	-	0.1	tr	tr	0.1	tr	0.1	0.1
90	*cis*-farnesol	1680	-	-	-	-	-	-	-	-	tr	-	-	-	-
91	β-sinensal	1686	-	0.1	-	-	-	-	-	-		-	-	tr	tr
92	pentadecanal	1711	-	-	-	-	-	-	0.1	-	-	-	-	-	-
93	mintsulfide	1731	-	-	-	-	-	-	-	-	-	-	-	tr	tr
94	6,10,14-trimethyl pendadecanone	1836	-	-	-	-	-	-	tr	-	-	-	-	-	-
95	E.E-farnesyl acetone	1904	-	-	-	-	-	-	tr	-	-	-	-	-	-
96	palmitic acid	1961	-	-	-	-	-	-	tr	-	-	-	-	-	-
**Total**			**99.7**	**99.8**	**99.9**	**99.5**	**99.7**	**99.2**	**95.7**	**99.9**	**99.7**	**98.9**	**99.9**	**99.1**	**99.2**

^a^ Retention indices were calculated against C9–C23 *n*-alkanes on the HP 5MS capillary column.
